# Thyroid Hormone-Induced Cytosol-to-Nuclear Translocation of Rat Liver Nrf2 Is Dependent on Kupffer Cell Functioning

**DOI:** 10.1100/2012/301494

**Published:** 2011-12-20

**Authors:** Luis A. Videla, Pamela Cornejo, Pamela Romanque, Catherine Santibáñez, Iván Castillo, Romina Vargas

**Affiliations:** ^1^Molecular and Clinical Pharmacology Program, Institute of Biomedical Sciences, Faculty of Medicine, University of Chile, Casilla 70000, Santiago-7, Chile; ^2^Faculty of Medicine, Diego Portales University, Santiago, Chile; ^3^School of Medicine, Faculty of Medicine, Catholic University of Maule, Talca, Chile

## Abstract

L-3,3′,5-triiodothyronine (T_3_) administration upregulates nuclear factor-E2-related factor 2 (Nrf2) in rat liver, which is redox-sensitive transcription factor mediating cytoprotection. In this work, we studied the role of Kupffer cell respiratory burst activity, a process related to reactive oxygen species generation and liver homeostasis, in Nrf2 activation using the macrophage inactivator gadolinium chloride (GdCl_3_; 10 mg/kg i.v. 72 h before T_3_ [0.1 mg/kg i.p.]) or NADPH oxidase inhibitor apocynin (1.5 mmol/L added to the drinking water for 7 days before T_3_), and determinations were performed 2 h after T_3_. T_3_ increased nuclear/cytosolic Nrf2 content ratio and levels of heme oxygenase 1 (HO-1), catalytic subunit of glutamate cysteine ligase, and thioredoxin (Western blot) over control values, proteins whose gene transcription is induced by Nrf2. These changes were suppressed by GdCl_3_ treatment prior to T_3_, an agent-eliciting Kupffer-cell depletion, inhibition of colloidal carbon phagocytosis, and the associated respiratory burst activity, with enhancement in nuclear inhibitor of Nrf2 kelch-like ECH-associated protein 1 (Keap1)/Nrf2 content ratios suggesting Nrf2 degradation. Under these conditions, T_3_-induced tumor necrosis factor-**α** (TNF-**α**) response was eliminated by previous GdCl_3_ administration. Similar to GdCl_3_, apocynin given before T_3_ significantly reduced liver Nrf2 activation and HO-1 expression, a NADPH oxidase inhibitor eliciting abolishment of colloidal carbon-induced respiratory burst activity without altering carbon phagocytosis. It is concluded that Kupffer cell functioning is essential for upregulation of liver Nrf2-signaling pathway by T_3_. This contention is supported by suppression of the respiratory burst activity of Kupffer cells and the associated reactive oxygen species production by GdCl_3_ or apocynin given prior to T_3_, thus hindering Nrf2 activation.

## 1. Introduction

Kupffer cells reside in liver sinusoids representing approximately 35% of hepatic nonparenchymal cells. These liver macrophages have scavenger receptors that are essential for eliminating blood borne bacteria [1]. In addition, activated Kupffer cells produce and release several mediators including cytokines, lipid substances, and reactive oxygen species (ROS), which can function locally or systemically to mediate immune responses [1]. These responses play a key role in the homeostatic adaptation to liver injury; however, if dysregulated, they can induce acute or chronic liver damage [2–4]. Cytotoxicity of Kupffer cells has been implicated in ischemia-reperfusion (IR) injury, an inflammatory model underlying drastic ROS generation [5], whereas preconditioning strategies against IR injury have been associated with moderate increases in ROS production [6, 7].

 Thyroid hormone- (L-3,3′,5-triiodothyronine, T_3_) induced calorigenic effects involving ROS generation in the liver has been proposed as a preconditioning mechanism for IR injury [8]. T_3_-induced ROS generation occurs at different subcellular sites of hepatocytes and in the respiratory burst of Kupffer cells, triggering the activation of the transcription factors nuclear factor-*κ*B (NF-*κ*B), signal transducer and activator of transcription 3 (STAT3), and activating protein 1 (AP-1). Under these conditions, the redox upregulation of Kupffer cell-dependent expression of cytokines (tumor necrosis factor-*α* (TNF-*α*), interleukin (IL)-1, and IL-6) is achieved, which upon interaction with specific receptors in hepatocytes trigger the expression of antioxidant enzymes (manganese superoxide dismutase, inducible nitric oxide synthase) [9, 10], antiapoptotic proteins (Bcl-2) [9], and acute-phase proteins (haptoglobin, *β*-fibrinogen) [11]. These responses, and the promotion of hepatocyte and Kupffer cell proliferation observed [12, 13], represent cytoprotective effects reestablishing redox homeostasis, promoting cell survival, and protecting the liver against IR injury [7].

Activation of nuclear factor-E2-related factor 2 (Nrf2) also affords cytoprotection, in addition to NF-*κ*B-, STAT3-, and AP-1-dependent signaling pathways, a transcription factor whose cytosol-to-nuclear translocation has been recently found to be triggered by T_3_ administration through a redox-dependent mechanism [14]. Nrf2 signaling is characterized by its sensitivity to low levels to ROS [15], controls the expression of antioxidant components, detoxification enzymes, membrane transporters, or 26 S proteasome components, and interplays with NF-*κ*B affording anti-inflammatory responses [16–19]. Thus, the cytoprotective effects of T_3_-induced liver Nrf2 activation may represent an alternate mechanism for liver preconditioning, a condition associated with Kupffer cell functioning that may constitute a new therapeutic option for liver surgery and liver transplantation in man using reduced-size grafts from living donors [7, 8, 20]. According to these considerations, the purpose of this study was to investigate whether T_3_-induced liver Nrf2 activation depends on the respiratory burst activity of Kupffer cells, a process related to ROS generation and liver homeostasis. For this purpose, Nrf2 activation, as assessed by cytosol-to-nuclear translocation, was determined in rat liver either without or with pretreatment with the Kupffer cell inactivator gadolinium chloride (GdCl_3_) [21] or with the NADPH oxidase inhibitor apocynin [22] prior to T_3_. These studies were correlated with the assessment of Kupffer cell functioning by means of colloidal carbon phagocytosis and the respective respiratory burst activity in perfused livers. The expression of the antioxidant proteins heme oxygenase-1 (HO-1), catalytic subunit of glutamate cysteine ligase (GCLC), and thioredoxin (Trx) were chosen as prototypical genes controlled by Nrf2 signaling, in addition to the levels of the Nrf2 inhibitor kelch-like ECH-associated protein 1 (Keap1) [17–19].

## 2. Methods

### 2.1. Animal Treatments

Male Sprague-Dawley rats (Animal Facility of the Institute of Biomedical Sciences, Faculty of Medicine, University of Chile) weighing 180–200 g, housed on a 12-h light/dark cycle, and fed with rat chow and water *ad libitum*, received a single intraperitoneal dose of 0.1 mg of T_3_/kg body weight or equivalent volumes of the hormone vehicle 0.1 N NaOH (controls). Kupffer cells were selectively eliminated by a single intravenous injection of 10 mg of GdCl_3_/kg body weight [21] given 72 h before T_3_ administration, and control animals received equivalent volumes of saline. A separate group of rats was given the selective NADPH oxidase inhibitor apocynin (1.5 mmol/L) added to the drinking water for 7 days prior to T_3_, a protocol shown to effectively inhibit NADPH oxidase activity in rats [22]. Studies were carried out 2 h after T_3_ administration in animals anesthetized with intraperitoneal (1 mL/kg) zolazepam chlorhydrate (25 mg/mL) and tiletamine chlorhydrate (25 mg/mL) (Zoletil-50; Virbac S/A, Carros, France). In the group of rats subjected to GdCl_3_-T_3_- combined treatment, levels of serum TNF-*α* were measured by ELISA (UltraSensitive Cytoscreen kit, Biosource International, Camarillo, CA, USA) according to manufacturer's specifications. Experimental animal protocols and animal procedures complied with the Guide for the Care and Use of Laboratory Animals (National Academy of Sciences, NIH Publication 86-23, revised 1985) and were approved by Ethics Committee of the Faculty of Medicine, University of Chile (CBA 0269 FMUCH).

### 2.2. Kupffer-Cell Inactivation

Liver slices were obtained in anesthetized (Zoletil-50) rats at 24 to 72 h after-GdCl_3_, and kinetic changes of ED2-immunolabelled Kupffer cells were determined by immunohistochemistry using a commercial kit (AbD Serotec, Oxford, UK). Briefly, liver samples were fixed in phosphate-buffered formalin (pH 7.4) and incubated with a primary mouse antibody to ED2, followed by incubation with biotin-conjugated secondary goat antibody. Positive reactions were visualized with 3,3'-diaminobenzidine, and results are expressed as the number of cells determined in 10 different 0.7 mm^2^ areas per liver from 3 rats per timepoint [23].

### 2.3. Liver Perfusion, Colloidal Carbon Uptake, and Carbon-Induced Respiratory Activity

Livers from animals anesthetized with Zoletil-50 were perfused with a solution containing 118 mM NaCl, 4.8 mM KCl, 1.2 mM KH_2_PO_4_, 1.2 mM MgSO_4_, 2.5 mM CaCl_2_, 25 mM NaHCO_3_, and 10 mM glucose, equilibrated with and O_2_/CO_2_ mixture (19 : 1, vol/vol) to give pH 7.4, through a cannula placed in the portal vein. Perfusion was carried out at constant flow rates (3.5 to 4 mL/g liver/min) and temperature (36 to 37°C), without recirculation of the perfusate [13, 24]. After 15 min equilibration of perfused livers, O_2_ consumption (QO_2_) was determined in the effluent perfusate collected via a cannula placed in the vena cava and allowed to flow through a Clark-type oxygen electrode. For determination of colloidal carbon uptake by perfused livers, suspensions of India ink (Rotring, Hamburg, Germany) were prepared, dialysed, and infused between 30 to 45 min of perfusion at the concentration of 0.5 mg/mL. Perfusate samples were taken every 10 min in the presence and absence of the liver to measure the absorbance of colloidal carbon at 623 nm [24] (specific extinction coefficient of 0.97 [mg/mL]^−1^) [13]. Rates of carbon uptake were calculated from influent minus effluent concentration differences, referred to the perfusion flow. The respiratory burst activity induced by colloidal carbon infusion was assessed by the integration of the area under the QO_2_ curves between 30 and 45 min, and expressed as *μ*mol O_2_/g liver [13]. These parameters were determined in control rats and in animals after 2 h of T_3_ administration and pretreatment for 72 h with GdCl_3_ or for 7 days with apocynin prior to T_3_.

### 2.4. Western Blot Analysis of Nrf2, Keap1, HO-1, GCLC, and Trx

Liver samples (100–500 mg) frozen in liquid nitrogen were homogenized and suspended in a buffer solution pH 7.9 containing 10 mM HEPES, 1 mM EDTA, 0.6% Nonidet P-40, 150 mM NaCl, and protease inhibitors (1 mM phenylmethylsulfonyl fluoride, 1 *μ*g/mL aprotinin, 1 *μ*g/mL leupeptin, and 1 mM orthovanadate). Nuclear protein extracts (100 *μ*g) and soluble protein fractions (60 *μ*g) were mixed with sample loading buffer pH 6.8 (2% SDS, 0.0625 M Tris, 10% glycerol, and 2.5% *β*-mercaptoethanol) and heated at 95°C for 5 min, separated on 12% polyacrylamide gels using SDS-PAGE [25], and transferred to nitrocellulose membranes [26], which were blocked for 1 hour at room temperature with TBS-containing 5% bovine serum albumin. The blots were washed with TBS containing 0.1% Tween 20 and hybridized with either rabbit polyclonal antibodies for Nrf2, Keap1, HO-1, GCLC, and Trx (Abcam, Cambridge, MA), or mouse monoclonal antibodies for *β*-actin (ICN Biomedicals, Inc., Aurora, OH) and lamin A/C (BD Transduction Laboratories, San José, CA, USA). In all determinations, anti-*β*-actin was used as internal control for cytosolic fractions, whereas antilamin A/C was employed as internal control for nuclear fractions. In addition, the membranes were stained with anti-*α*-tubulin or anti-lamin A/C to confirm contamination of the cytosolic and nuclear fractions. After extensive washing, the antigen-antibody complexes were detected using horseradish peroxidase goat anti-rabbit IgG or goat anti-mouse IgG and a SuperSignal West Pico Chemiluminescence kit detection system (Pierce, Rockford, IL, USA). Bands were quantified by densitometry using Scion Image (Scion Corp., Frederick, MD).

### 2.5. Statistics

Data showing Gaussian distribution according to the Kolmogorov-Smirnov test are expressed as means ± SEM for the number of separate experiments indicated. One-way or two-way ANOVA and the Newman-Keuls^,^ test or Student's *t*-test for unpaired data assessed the statistical significance (*P* < 0.05) of differences between mean values as indicated. All statistical analyses were computed employing GraphPad Prism^TM^ version 2.0 (GraphPad Software Inc., San Diego, CA, USA).

## 3. Results

**Figure 1:**
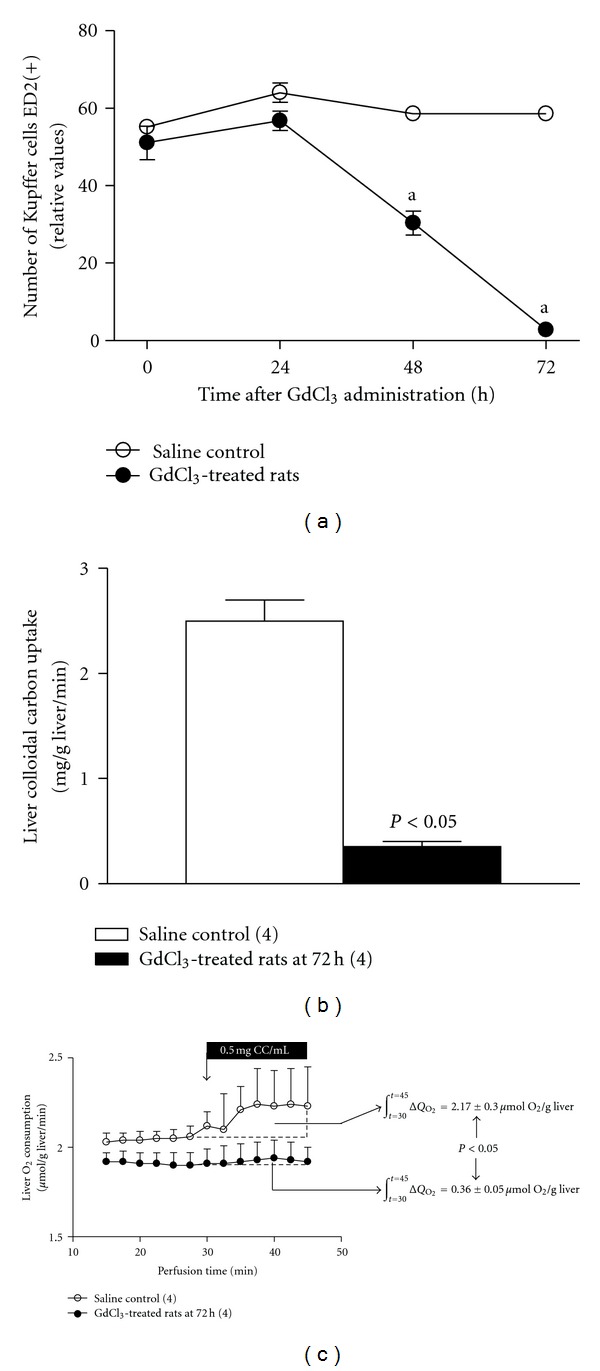
Gadolinium chloride (GdCl_3_) administration is associated with suppression of Kupffer cell functioning in rat liver. Kinetics of Kupffer cell inactivation after GdCl_3_ treatment (time zero) in livers from euthyroid rats by immunohistochemistry using ED2 antibody (a), rate of colloidal carbon uptake (b), and carbon-induced liver respiratory activity (ΔQO_2_) (c) assessed in isolated perfused livers at 72 h after GdCl_3_ treatment. ΔQO_2_ was calculated by integration of the area under the O_2_ consumption curves between 30 and 45 min perfusion (c). Values shown represent means ± SEM for 4 rats per experimental group. ^a^ *P* < 0.05 versus controls assessed by Student's *t*-test for unpaired data.

Administration of the Kupffer cell inactivator GdCl_3_ to euthyroid rats elicited a decrease in the number of ED2(+) cells, with 95% (*P* < 0.05) depletion observed at 72 h (Figure 1(a)), as assessed by immunohistochemical technique with ED2 antibody. Studies using the isolated perfused liver revealed that, at 72 h after treatment, GdCl_3_ reduced by 86% and 83% (*P* < 0.05) the rate of colloidal carbon uptake (Figure 1(b)) and the associated carbon-induced respiratory activity (Figure 1(c)), respectively, compared to liver perfusions in the absence of carbon infusion. According to these results, the influence of Kupffer cells on T_3_-induced liver Nrf2 activation was studied by giving T_3_ at the time of maximal ED2(+) Kupffer-cell inactivation (72 h after GdCl_3_), and studies on T_3_ action were carried out 2 h after T_3_ administration, time at which Nrf2 activation is attained [14].

**Figure 2:**
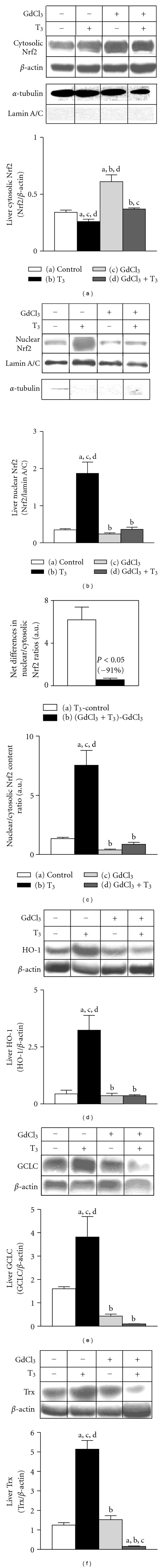
Gadolinium chloride (GdCl_3_) administration is associated with suppression of T_3_-induced activation of liver Nrf2 signaling. Determinations were performed at 2 h after T_3_ administration in rats pretreated with GdCl_3_ for 72 h. (a) Levels of cytosolic Nrf2 protein (68 kDa), *β*-actin (43 kDa), *α*-tubulin (52 kDa), and lamin A/C (65 kDa); (b) levels of nuclear Nrf2 protein, lamin A/C, and *α*-tubulin; (c) nuclear/cytosolic Nrf2 content ratios and net effects of T_3_ and GdCl_3_ treatments (*inset*); (d) levels of heme oxygenase 1 (HO-1) protein (33 kDa); (e) levels of catalytic subunit of glutamate cysteine ligase (GCLC) protein (73 kDa); (f) levels of thioredoxin (Trx) protein (12 kDa). Values shown represent means ± SEM for 3 to 6 rats per experimental group. Significance (*P* < 0.05; two-way ANOVA and the Newman-Keuls' test) is indicated by the letters identifying each experimental group. Significance in the inset of (c) was calculated by Student's *t*-test for unpaired data.

**Figure 3:**
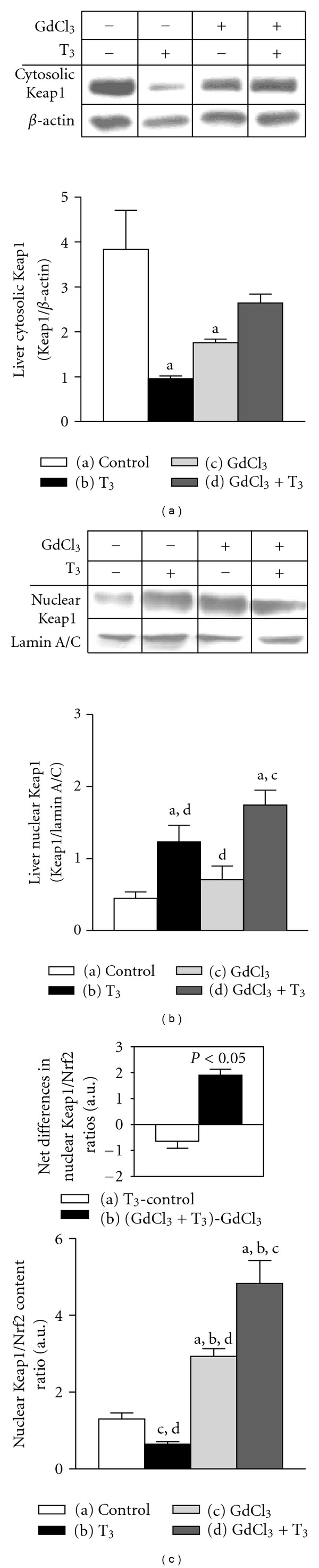
Gadolinium chloride (GdCl_3_) administration is associated with enhancement of liver Keap1/Nrf2 ratios over values in T_3_-treated rats. Determinations were performed at 2 h after T_3_ administration in rats pretreated with GdCl_3_ for 72 h. (a) Levels of cytosolic Keap1 protein (70 kDa) and *β*-actin (43 kDa); (b) levels of nuclear Keap1 protein and lamin A/C (65 kDa); (c) nuclear Keap1/Nrf2 content ratios and net effects of T_3_ and GdCl_3_ treatments (*inset)*. Values shown represent means ± SEM for 4 to 6 rats per experimental group. Significance (*P* < 0.05; two-way ANOVA and the Newman-Keuls' test) is indicated by the letters identifying each experimental group. Significance in the inset of (c) was calculated by Student's *t*-test for unpaired data.

Liver Nrf2 activation induced at 2 h after T_3_ administration was evidenced by the significant 24% decrease in the content of cytosolic Nrf2 (Figure 2(a)) and 434% enhancement in that of nuclear Nrf2 (Figure 2(b)), with a 463% increase in nuclear/cytosolic Nrf2 ratio (Figure 2(c)). Treatment with GdCl_3_ did not significantmodifiy the liver nuclear/cytosolic Nrf2 ratio when given alone (Figure 2(c)). However, cytosolic and nuclear Nrf2 levels after combined GdCl_3_-T_3_ protocol were comparable to control values (Figures 2(a) and 2(b)), leading to a net 91% decrease (*P* < 0.05) in the nuclear/cytosolic Nrf2 ratio compared to the net effect of T_3_ alone (Figure 2(c), inset). Under these conditions, upregulation of liver HO-1 (Figure 2(d)), GCLC (Figure 2(e)), and Trx (Figure 2(f)) by T_3_ was suppressed by the combined GdCl3-T_3_ treatment, without significant effects of GdCl_3_ when given alone (Figures 2(d), 2(e) and 2(f)). These findings were observed concomitantly with 7.5-fold increase in the serum TNF-*α* levels by T_3_, with a net 92% diminution being elicited by the combined GdCl_3_-T_3_ treatment (a) control, 2 ± 1 (*n* = 9) pg TNF-*α*/mL; (b) T_3_, 15 ± 1 (*n* = 3); (c) GdCl_3_, 3 ± 2 (*n* = 3); (d) GdCl_3_-T_3_, 4 ± 2 (*n* = 3); (b) versus (a), (c), and (d), *P* < 0.05). Furthermore, liver Nrf2 inhibitor Keap1 levels in the cytosol exhibited 75% reduction in T_3_-treated rats over controls (Figure 3(a)), whereas those of nuclear Keap1 were enhanced by 173% (Figure 3(b)), without significant changes in nuclear Keap1/Nrf2 ratios (Figure 3(c)). Net differences in the latter parameter indicate a substantial enhancement (*P* < 0.05) in animals subjected to combined GdCl_3_-T_3_ treatment [(GdCl_3_ + T_3_) − GdCl_3_] compared to rats given T_3_ alone [T_3 _− control] (Figure 3(c), inset).

**Figure 4:**
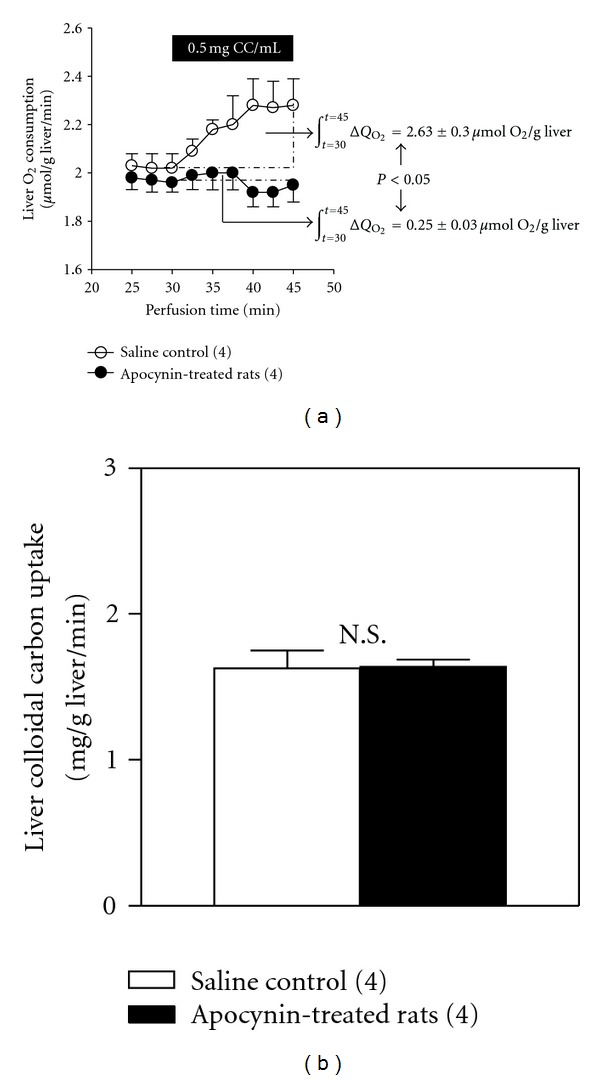
Apocynin administration is associated with suppression of colloidal carbon-induced liver respiratory activity (ΔQO_2_) assessed in isolated perfused livers from euthyroid rats (a), without altering carbon phagocytosis (b). Determinations were carried out 7 days after apocynin treatment. ΔQO_2_ was calculated by integration of the area under the O_2_ consumption curves between 30 and 45 min perfusion. Values shown represent means ± SEM for 4 rats per experimental group. Significance (*P* < 0.05) was calculated by Student's *t*-test for unpaired data.

**Figure 5:**
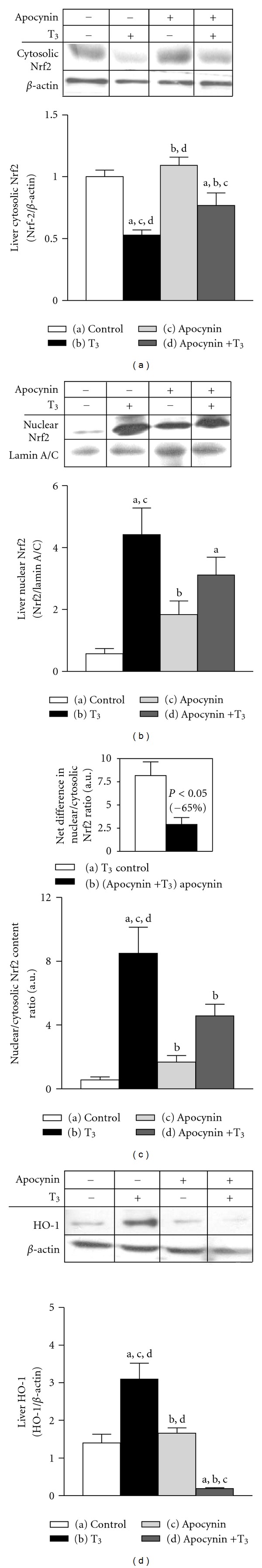
Apocynin administration is associated with diminution of T_3_-induced activation of liver Nrf2 signaling. Determinations were performed at 2 h after T_3_ administration in rats pretreated with apocynin for 7 days. (a) Levels of cytosolic Nrf2 protein (68 kDa) and *β*-actin (43 kDa); (b) levels of nuclear Nrf2 protein and lamin A/C (65 kDa); (c) nuclear/cytosolic Nrf2 content ratios and net effects of T_3_ and apocynin treatments (*inset*); (d) levels of heme oxygenase 1 (HO-1) protein (33 kDa). Values shown represent means ± SEM for 3 to 4 rats per experimental group. Significance (*P* < 0.05; two-way ANOVA and the Newman-Keuls' test) is indicated by the letters identifying each experimental group. Significance in the inset of (c) was calculated by Student's *t*-test for unpaired data.

Administration of apocynin to euthyroid rats resulted in 90% decrease (*P* < 0.05) in carbon-induced respiratory burst activity assessed in liver perfusion studies (Figure 4(a)), without significant changes in the rate of colloidal carbon uptake (Figure 4(b)). Liver Nrf2 activation by T_3_ administration involved significant 48% decrease in the content of cytosolic Nrf2 (Figure 5(a)), 675% enhancement in nuclear Nrf2 levels (Figure 5(b)), and 14.9-fold increase in nuclear/cytosolic Nrf2 ratio (Figure 5(c)) over control values. Apocynin administration prior to T_3_ elicited 29% diminution (*P* < 0.05) in cytosolic Nrf2 (Figure 5(a)), with comparable values of nuclear Nrf2 (Figure 5(b)) and nuclear/cytosolic Nrf2 ratios (Figure 5(c)) to those in rats given apocynin alone, thus eliciting a net 65% reduction in nuclear/cytosolic Nrf2 ratios (Figure 5(c), inset). Under these conditions, T_3_-induced upregulation of liver HO-1 levels was suppressed in apocynin-T_3_-treated animals (Figure 5(d)).

## 4. Discussion

Kupffer cell functioning assessed in the isolated perfused rat liver by means of colloidal carbon infusion allows the continuous estimation of the associated rate of carbon-particle phagocytosis and the respiratory burst activity of liver macrophages [13, 24]. The use of this model system provided evidence for the role of Kupffer cells in the hepatotoxicity of lindane [27], acetaminophen [28], and copper [29], as well as Kupffer cell function adaptation leading to hepatoprotection after T_3_ administration [13, 20]. Data reported in this study indicate that T_3_ administration up-regulates liver Nrf2 signaling depending on Kupffer cell functioning. Early (2 h) liver Nrf2 activation triggered by T_3_ treatment evidenced by 4.63-fold enhancement in nuclear/cytosolic Nrf2 ratios denoting cytosol-to-nuclear Nrf2 translocation, occurred without significant changes in nuclear Keap1/Nrf2 ratios. These data indicate that T_3_ achieves liver Nrf2 upregulation in a time interval at which Nrf2-dependent induction of its inhibitor Keap1 [30] does not occur, resulting in significant increases in the expression of the target genes controlled by Nrf2, namely, HO-1, GCLC, and Trx [18]. T_3_-induced liver Nrf2 activation involves a redox-dependent mechanism, considering that cytosol to nuclear Nrf2 translocation is blocked by N-acetylcysteine pretreatment [14]. The redox activation of Nrf2 is associated with ROS produced due to acceleration of liver O_2_ consumption by the calorigenic action of T_3_ exerted on hepatocytes and Kupffer cells [7], but it also may involve ROS generated in the respiratory burst activity of hepatic macrophages [13]. The latter proposal underlies redox activation of NF-*κ*B in Kupffer-cell of T_3_-treated animals [31], with consequent expression and release of TNF-*α*, as reported in this study. Interaction of TNF-*α* with TNF-*α* receptor-1 in hepatocytes may trigger mitochondrial ROS production [32, 33], reinforcing that achieved by actions of T_3_ on hepatocyte energy metabolism. Under these conditions, Nrf2 activation may be achieved by direct action of ROS [17–19] or through ROS-dependent formation of cyclopentenone-containing J isoprostanes from polyunsaturated fatty acids, which release Nrf2 upon binding to Keap1 [34, 35]. In addition, increased formation of Nrf2/c-Jun complexes may occur due to the ability of TNF-*α* to induce c-Jun nuclear-binding activity [36], heterodimerization that is required for ARE-mediated transcriptional activation [37].

 Dependency of T_3_-induced liver Nrf2 upregulation on Kupffer cells was demonstrated by inactivation of liver macrophages by GdCl_3_ [21] or inhibition of Kupffer-cell NADPH oxidase activity by apocynin [22, 38]. Administration of the GdCl_3_ 72 h prior to T_3_ achieved almost complete elimination of ED2(+) cells, a Kupffer cell subpopulation characterized with a ED2 antibody recognizing a membrane antigen of resident macrophages such as Kupffer cells [39, 40]. Liver ED2(+) cells are described as mature macrophages [41], which are mainly located in periportal areas of the liver lobule [42]. These mature liver macrophages have higher lysosomal enzyme activities, phagocytic capacity, and production of TNF-*α*, interleukin-1 and prostaglandin E_2_ than smaller ED1(+) cells located in midzonal and central areas [1], a subpopulation of liver macrophages that is not modified by GdCl_3_ administration [20]. Under conditions of GdCl_3_-induced Kupffer-cell depletion, activation of Nrf2 and expression of HO-1, GCLC, and Trx by T_3_ were abolished, concomitantly with significant enhancement in nuclear Keap1/Nrf2 ratios. The latter finding suggests that the nuclear abundance of Keap1 is increased by combined GdCl_3_-T_3_ treatment, which may allow an efficient nuclear export mechanism to terminate T_3_-induced Nrf2 signaling [43]. In agreement with these views, T_3_-induced TNF-*α* response is abolished in rats subjected to combined GdCl_3_-T_3_ treatment, which may suppress Kupffer cell-dependent TNF-*α*-induced mitochondrial ROS component, otherwise altering Keap1 to a form which does not have anti-Nrf2 effects [19]. The role of Kupffer cells in T_3_-induced liver Nrf2 activation suggested by hepatic macrophage depletion after GdCl_3_ administration is further supported by experiments using apocynin prior to T_3_. Apocynin inhibits the assembly of the ROS generator NADPH oxidase in neutrophils and macrophages after metabolic conversion, without altering phagocytosis, in addition to its free-radical scavenging properties [38]. In addition, Kupffer cell activation by hepatic IR upregulates kidney Nrf2 signaling to avoid remote organ dysfunction, as treatment with GdCl_3_ prior to liver IR attenuates the TNF-*α* response induced, reducing the enhancement in renal levels of the Nrf2 activator 15-deoxy-Δ^12,14^-protaglandin J_2_, Nrf2 nuclear translocation, and HO-1 expression [34].

## 5. Conclusion

Data presented suggest that Kupffer cell functioning is essential for upregulation of liver Nrf2 stress response-signaling pathway by T_3_. This is demonstrated by the abolishment of Nrf2 activation and Nrf2-dependent expression of HO-1, GCLC, and Trx by GdCl_3_-induced Kupffer cell depletion and apocynin inhibition of macrophage NADPH oxidase, when given prior to T_3_. These agents abrogate the respiratory burst activity of Kupffer cells and the associated ROS production, thus hindering Nrf2 activation. Data presented and the significant diminution in T_3_-induced changes in hepatic oxidative stress-related parameters by GdCl_3_ pretreatment, namely, reduced glutathione depletion and enhancement in lipid peroxidation and in the biliary glutathione disulfide release [13, 44], point to the crucial role of Kupffer cell functioning in mediating T_3_-dependent cytoprotection in the liver. This is understood in terms of T_3_-induced Kupffer cell-dependent development of a suitable prooxidant status in hepatocytes triggering the activation of protective, redox-sensitive transcription factors such as Nrf2, as well as NF-*κ*B, STAT3, and AP-1 [7].

## References

[1] Bilzer M, Roggel F, Gerbes AL (2006). Role of Kupffer cells in host defense and liver disease. *Liver International*.

[2] Jaeschke H (2011). Reactive oxygen and mechanisms of inflammatory liver injury: present concepts. *Journal of Gastroenterology and Hepatology*.

[3] Kolios G, Valatas V, Kouroumalis E (2006). Role of Kupffer cells in the pathogenesis of liver disease. *World Journal of Gastroenterology*.

[4] Tsukamoto H (2002). Redox regulation of cytokine expression in Kupffer cells. *Antioxidants and Redox Signaling*.

[5] Jaeschke H, Farhood A (1991). Neutrophil and Kupffer cell-induced oxidant stress and ischemia-reperfusion injury in rat liver. *American Journal of Physiology*.

[6] Casillas-Ramírez A, Mosbah IB, Ramalho F, Roselló-Catafau J, Peralta C (2006). Past and future approaches to ischemia-reperfusion lesion associated with liver transplantation. *Life Sciences*.

[7] Videla LA (2010). Hormetic responses of thyroid hormone calorigenesis in the liver: association with oxidative stress. *IUBMB Life*.

[8] Fernández V, Castillo I, Tapia G (2007). Thyroid hormone preconditioning: protection against ischemia-reperfusion liver injury in the rat. *Hepatology*.

[9] Fernández V, Tapia G, Varela P (2005). Redox up-regulated expression of rat liver manganese superoxide dismutase and Bcl-2 by thyroid hormone is associated with inhibitor of *κ*B-*α* phosphorylation and nuclear factor-*κ*B activation. *Journal of Endocrinology*.

[10] Fernández V, Tapia G, Varela P, Videla LA (2005). Redox regulation of thyroid hormone-induced Kupffer cell-dependent I*κ*B-*α* phosphorylation in relation to inducible nitric oxide synthase expression. *Free Radical Research*.

[11] Tapia G, Fernández V, Pino C, Ardiles R, Videla LA (2006). The acute-phase response of the liver in relation to thyroid hormone-induced redox signaling. *Free Radical Biology and Medicine*.

[12] Fernández V, Reyes S, Bravo S (2007). Involvement of Kupffer cell-dependent signaling in T_3_-induced hepatocyte proliferation *in vivo*. *Biological Chemistry*.

[13] Tapia G, Pepper I, Smok G, Videla LA (1997). Kupffer cell function in thyroid hormone-induced liver oxidative stress in the rat. *Free Radical Research*.

[14] Romanque P, Cornejo P, Valdés S, Videla LA (2011). Thyroid hormone administration induces rat liver Nrf2 activation: suppression by N-acetylcysteine pretreatment. *Thyroid*.

[15] Gloire G, Legrand-Poels S, Piette J (2006). NF-*κ*B activation by reactive oxygen species: fifteen years later. *Biochemical Pharmacology*.

[16] Kensler TW, Wakabayashi N, Biswal S (2007). Cell survival responses to environmental stresses via the Keap1-Nrf2-ARE pathway. *Annual Review of Pharmacology and Toxicology*.

[17] Kobayashi M, Yamamoto M (2005). Molecular mechanisms activating the Nrf2-Keap1 pathway of antioxidant gene regulation. *Antioxidants and Redox Signaling*.

[18] Singh S, Vrishni S, Singh BK, Rahman I, Kakkar P (2010). Nrf2-ARE stress response mechanism: a control point in oxidative stress-mediated dysfunctions and chronic inflammatory diseases. *Free Radical Research*.

[19] Zhang DD (2006). Mechanistic studies of the Nrf2-Keap1 signaling pathway. *Drug Metabolism Reviews*.

[20] Tapia G, Santibáñez C, Farías J (2010). Kupffer-cell activity is essential for thyroid hormone rat liver preconditioning. *Molecular and Cellular Endocrinology*.

[21] Hardonk MJ, Dijkhuis FWJ, Hulstaert CE, Koudstaal J (1992). Heterogeneity of rat liver and spleen macrophages in gadolinium chloride-induced elimination and repopulation. *Journal of Leukocyte Biology*.

[22] Gracia-Sancho J, Laviña B, Rodríguez-Vilarrupla A (2007). Evidence against a role for NADPH oxidase modulating hepatic vascular tone in cirrhosis. *Gastroenterology*.

[23] Gomes LF, Lorente S, Simon-Giavarotti KA, Areco KN, Araújo-Peres C, Videla LA (2004). Tri-iodothyronine differentially induces Kupffer cell ED1/ED2 subpopulations. *Molecular Aspects of Medicine*.

[24] Cowper KB, Currin RT, Dawson TL, Lindert KA, Lemasters JJ, Thurman RG (1990). A new method to monitor Kupffer-cell function continuously in the perfused rat liver. *Biochemical Journal*.

[25] Laemmli UK (1970). Cleavage of structural proteins during the assembly of the head of bacteriophage T4. *Nature*.

[26] Towbin H, Staehelin T, Gordon J (1979). Electrophoretic transfer of proteins from polyacrylamide gels to nitrocellulose sheets: procedure and some applications. *Proceedings of the National Academy of Sciences of the United States of America*.

[27] Videla LA, Troncoso P, Arisi ACM, Junqueira VBC (1997). Dose-dependent effects of acute lindane treatment on Kupffer cell function assessed in the isolated perfused rat liver. *Xenobiotica*.

[28] Tapia G, Cornejo P, Ferreira J, Fernández V, Videla LA (1997). Acetaminophen-induced liver oxidative stress and hepatotoxicity: influence of Kupffer cell activity assessed in the isolated perfused rat liver. *Redox Report*.

[29] Sans J, Aguilera AM, Faúndez P, Troncoso P, Fernández V, Videla LA (1999). Influence of copper-(II) on colloidal carbon-induced Kupffer cell-dependent oxygen uptake in rat liver: relation to hepatotoxicity. *Free Radical Research*.

[30] Lee OH, Jain AK, Papusha V, Jaiswal AK (2007). An auto-regulatory loop between stress sensors INrf2 and Nrf2 controls their cellular abundance. *Journal of Biological Chemistry*.

[31] Tapia G, Fernández V, Varela P, Cornejo P, Guerrero J, Videla LA (2003). Thyroid hormone-induced oxidative stress triggers nuclear factor-*κ*B activation and cytokine gene expression in rat liver. *Free Radical Biology and Medicine*.

[32] Gudz TI, Tserng KY, Hoppel CL (1997). Direct inhibition of mitochondrial respiratory chain complex III by cell-permeable ceramide. *Journal of Biological Chemistry*.

[33] Schulze-Osthoff K, Bakker AC, Vanhaesebroeck B, Beyaert R, Jacob WA, Fiers W (1992). Cytotoxic activity of tumor necrosis factor is mediated by early damage of mitochondrial functions. Evidence for the involvement of mitochondrial radical generation. *Journal of Biological Chemistry*.

[34] Tanaka Y, Maher JM, Chen C, Klaassen CD (2007). Hepatic ischemia-reperfusion induces renal heme oxygenase-1 via NF-E2-related factor 2 in rats and mice. *Molecular Pharmacology*.

[35] Gao L, Wang J, Sekhar KR (2007). Novel n-3 fatty acid oxidation products activate Nrf2 by destabilizing the association between Keap1 and Cullin3. *Journal of Biological Chemistry*.

[36] Yang H, Magilnick N, Ou X, Lu SC (2005). Tumour necrosis factor *α* induces co-ordinated activation of rat GSH synthetic enzymes via nuclear factor *κ*B and activator protein-1. *Biochemical Journal*.

[37] Kaspar JW, Niture SK, Jaiswal AK (2009). Nrf2:INrf2 (Keap1) signaling in oxidative stress. *Free Radical Biology and Medicine*.

[38] Stefanska J, Pawliczak R (2008). Apocynin: molecular aptitudes. *Mediators of Inflammation*.

[39] Damoisequx JG, Dopp EA, Calame W, Chao D, MacPherson GG, Dijkstra CD (1994). Rat macrophage lysosomal membrane antigen recognized by monoclonal antibody ED1. *Immunology*.

[40] Ide M, Yamate J, Machida Y (2003). Emergence of different macrophage populations in hepatic fibrosis following thioacetamide-induced acute hepatocyte injury in rats. *Journal of Comparative Pathology*.

[41] Armbrust T, Ramadori G (1996). Functional characterization of two different Kupffer cell populations of normal rat liver. *Journal of Hepatology*.

[42] Bouwens L, Baekeland M, de Zanger R, Wisse E (1986). Quantitation, tissue distribution and proliferation kinetics of Kupffer cells in normal rat liver. *Hepatology*.

[43] Jain AK, Bloom DA, Jaiswal AK (2005). Nuclear import and export signals in control of Nrf2. *Journal of Biological Chemistry*.

[44] Fernández V, Videla LA, Tapia G, Israel Y (2002). Increases in tumor necrosis factor-*α* in response to thyroid hormone-induced liver oxidative stress in the rat. *Free Radical Research*.

